# Jahn–Teller distortion in Sr_2_FeO_4_: group-theoretical analysis and hybrid DFT calculations

**DOI:** 10.1038/s41598-023-43381-7

**Published:** 2023-09-30

**Authors:** Guntars Zvejnieks, Yuri Mastrikov, Denis Gryaznov

**Affiliations:** Institute of Solid State Physics, University of Latvia, Kengaraga Str. 8, Riga, 1063 Latvia

**Keywords:** Structure of solids and liquids, Electronic structure, Materials science, Computational methods

## Abstract

We present theoretical justification for distorted Ruddlesden–Popper (RP) phases of the first-order by using hybrid density functional theory (DFT) calculations and group-theoretical analysis. We, thus, demonstrate the existence of the Jahn–Teller effect around an Fe$^{4\texttt {+}}$ ion in Sr$_{2}$FeO$_{4}$. On the calculation side, we have established a combination of Wu–Cohen (WC) exchange and Perdew-Wang (PW) correlation in a three-parameter functional WC3PW, giving the most accurate description of Sr$_{2}$FeO$_{4}$ from the comparison of three hybrid DFT functionals. Self-consistently obtained Hartree–Fock exact exchange of 0.16 demonstrates consistent results with the experimental literature data. Importantly, we explain conditions for co-existing proper and pseudo-Jahn–Teller effects from the crystalline orbitals, symmetry-mode analysis and irreps products. Moreover, phonon frequency calculations support and confirm the results of symmetry-mode analysis. In particular, the symmetry-mode analysis identifies a dominating irreducible representation of the Jahn-Teller mode (X2+) and corresponding space group (SG) of ground state structure (SG *Cmce* model). Therefore, the usually suggested high-symmetry tetragonal crystal structure (SG *I*4/*mmm* model) is higher in energy by 121 meV/f.u. (equivalent to the Jahn-Teller stabilization energy) compared with the distorted low-symmetry structure (SG *Cmce* model). We also present diffraction patterns for the two crystal symmetries to discuss the differences. Therefore, our results shed light on the existence of low-symmetry RP phases and make possible direct comparisons with future experiments.

## Introduction

Discovered in the 1950s in pioneering works for perovskite SrTiO$_{3}$ structures, i.e. Sr$_{2}$TiO$_{4}$^1^ and Sr$_{3}$Ti$_{2}$O$_{7}$^2^, materials able to exist in the Ruddlesden–Popper (RP) structure (A$_{n\texttt {+}1}$B$_{n}$O$_{3n\texttt {+}1}$) are experiencing growing interest due to high technological relevance. An intriguing interplay between behaviours of perovskite (ABO$_{3}$) and rock salt (AO) layers (phases) in the RP structure lies at the heart of this growing interest. Developments in this direction led to recent experiments on the synthesis and growth procedures for the RP structures with the SrTiO$_{3}$ phases for $n{\ge }20$^3^ and with the LaNiO$_{3}$ phases with *n* up to 5^4^. So, a fundamental understanding of mechanisms controlling RP structures’ properties is still the highest priority.

On-going active research around these materials is inspired by fascinating effects of spin, charge and orbital ordering typically demonstrated by perovskites with a transition metal B-cation in the ABO$_{3}$-structure^5^ and by layered transition metal oxides in the ABO$_{2}$-structure^6^. All these effects are accompanied and even further pronounced by the Jahn–Teller effect playing, thus, a very significant role. Below, we call the Jahn–Teller effect if the vibronic modes are not studied in detail. Otherwise, we specify proper Jahn–Teller or pseudo-Jahn–Teller effect^7^. For example, the Jahn–Teller effect is observed around Fe$^{4\texttt {+}}$ ions in distorted monoclinic La$_{0.5}$Sr$_{0.5}$FeO$_{3}$^8^ using hybrid density functional theory (DFT) calculations and in oxidized SrFe$_{x}$Ti$_{1-x}$O$_{3}$ using XAS and vibrational spectroscopy^9^. On the other hand, La$_{0.33}$Sr$_{0.67}$FeO$_{3-\delta }$ experiences charge disproportionation of Fe$^{4\texttt {+}}$ into Fe$^{3\texttt {+}}$ and Fe$^{5\texttt {+}}$^10^ whereas a parent SrFeO$_{3-\delta }$ is a metallic compound with the helical magnetic ordering^11^. The oxygen vacancies play an essential role in these two materials, confirmed by experiments^12,13^ and DFT calculations^14^.

In the present study, we focus on the Fe-based system, Sr$_{2}$FeO$_{4}$ (SFO). According to experiments^15^, SFO is a negative charge transfer insulator^16,17^. If so, then its similarities with BaFeO$_{3}$^18^ and BaCoO$_{3}$^19^ may be expected. As is shown by us in Refs.^20,21^, the oxygen 2*p* electrons contribute mainly to the Fermi energy giving rise to delocalised holes on the oxygen sub-lattice and metallic states in fully oxidised BaFeO$_{3}$ and BaCoO$_{3}$. On the other hand, BaCoO$_{3}$ undergoes the Jahn–Teller distortion and the band gap opening^21^. BaFeO$_{3}$ also demonstrates the presence of the Jahn-Teller effect, which becomes negligible under doping with Sr^22^. Notice that BaFeO$_{3}$ is successfully analysed in an experimental study^18^ employing several methods, namely HAXPES, XAS and XMCD, in combination with the configuration interaction cluster-model to accurately show that the ground state is $3d^{5}\underline{L}$ ($\underline{L}$: ligand hole) indicating holes on the oxygen sublattice. Finally, these extensive experiments also indicate a small band gap in BaFeO$_{3}$. Furthermore, recent DFT+U studies^22,23^ confirm this electronic structure for BaFeO$_{3-\delta }$ and present deeper insight into its orbital nature.

Below the Néel temperature of $T_N{=}56$ K, SFO orders antiferromagnetically, where spins adopt elliptical cycloidal magnetic structure with modulated magnetic moments between 1.9 and 3.5 $\mu _B$^15^. However, the spin-flop transition is observed with an increasing magnetic field between 3 and 6 T. Finally, with increasing pressure between 5 and 8 GPa, the spin spiral transforms into a ferromagnetic structure.

SFO consists of the inter-grown single rock salt layer (SrO-layer) with a single ($n{=}1$ in Sr$_{n\texttt {+}1}$Fe$_{n}$O$_{3n\texttt {+}1}$) perovskite layer (FeO$_2$-layer)^24^ and comprises a tetravalent Fe in the high electronic spin state $3d^4$: $t_{2g}^3e_g^1$^15,25^. The crystal structure of SFO powder is indexed using the tetragonal space group *I*4/*mmm*^25^ in a broad temperature range. However, the elliptically cycloidal spiral spin structure is incompatible with the *I*4/*mmm* space group (SG), and lower symmetry is expected^15^. Since Fe occupies 2a Wyckoff position in the *I*4/*mmm* SG, only the ferromagnetic structure can be considered in the derived magnetic SG $I4/mm'm'$. A hidden distorted structure, invisible to standard diffraction techniques, could also explain the insulating ground state of SFO^15^. Notably, very recent experimental findings by Jiang et al.^26^ for a series La$_{1.9}$BO$_{4\texttt {+}\delta }$ where B is a mixture of Mg, Cu, Co, Ni, Zn suggest symmetry lowering. Besides the BO$_{6}$ octahedra distortion, they also identify the space group *Cmce* (SG 64) as the low (room) temperature phase. Previous experimental studies of Midouni et al.^27^ and Niwa et al.^28^ indicate the structural phase transition from the tetragonal to orthorhombic phase for La$_{2-x}$Cu$_{x}$CaO$_{4-\delta }$, Nd$_{2}$NiO$_{4+\delta }$ and Pr$_{2}$NiO$_{4+\delta }$, respectively, depending on the A-cation and $\delta$. Also, some papers based on the DFT calculations mention low symmetry structures for the perfect bulk crystal of (La$_{1-x}$Sr$_{x}$)$_{2}$MO$_{4}$^29^ (only the SG 64 is mentioned in Supporting Information^29^ without a detailed discussion of the symmetry reduction mechanism) and Sr$_{3}$Fe$_{2}$O$_{7-\delta }$^30^ (the Jahn-Teller effect is partially discussed for $\delta <1$ without SG specification).

There is another interesting example in the literature when experimental studies show the presence of orthorhombic SG, but theoretical studies try to explain its stabilization from different viewpoints. These experimental studies concern Cu$^{2+}$-containng K$_{2}$CuF$_{4}$ (SG *Bbcm*, non-standard setting of SG 64)^31^ and Rb$_{2}$CuCl$_{4}$ (SG *Cmca*, standard setting of SG 64)^32^. García-Fernández et al.^33^ and Aramburu et al.^34^ point out the role of internal electric field of the rest of the crystal around the CuL$_{6}$ complexes, where L: F or Cl, in K$_{2}$CuF$_{4}$ and Rb$_{2}$CuCl$_{4}$. Interestingly, the former crystal is insulating, whereas the latter one is half-metallic in the high symmetry tetragonal phase^35^. Through the calculation of electrostatic potentials for the Cu-L bonds in these materials, the localization of electrons in the xy-plane is established, i.e. on the 3d$_{x^2-y^2}$ orbital (which is independent of the crystal symmetry employed^33,34^), giving rise to a specific orbital ordering. They also discuss an additional orthorhombic instability but do not associate it with the Jahn–Teller distortion. Liu et al. ^35^ discussed the formation of rhombuses due to the Jahn–Teller effect in the xy-plane in the low symmetry orthorhombic Rb$_{2}$CuCl$_{4}$ which is in line with the present study.

Our calculation results show the orthorhombic SG 64 for SFO, too. Below, we discuss the underlying mechanisms of this structural distortion in SFO with the help of group-theoretical considerations and hybrid DFT calculations. In particular, the latter is needed to properly treat exchange-correlation effects in such systems as SFO. Thus, the hybrid DFT calculations are based on the LCAO method and Gaussian basis set approach implemented in CRYSTAL computer code^36,37^. The presented results might be relevant for all previously obtained structural tetragonal-orthorhombic phase transitions in the RP-phase of $n{=}1$. We emphasise that group-theoretical and symmetry analysis is necessary to identify the distorted structure’s final space group and analyse the crystalline orbital formation and properties.

## Group-theoretical analysis

### Symmetry-mode analysis

The primary goal of our approach is to find a distortion pattern of the FeO$_{6}$ octahedra and, thus, its symmetry reduction. At the initial stage of our calculations, a complete symmetry switch-off applied to the supercell created from the high symmetry structure (SG *I*4/*mmm* model) leads to geometry relaxation without any symmetry constraints (SG *P*1 model). We then rely on the symmetry-mode analysis and AMPLIMODES program^38,39^ available on Bilbao Crystallographic Server (BCS)^40^ to identify the remaining symmetry (if any) in the calculated supercell relative to the parent – high symmetry *I*4/*mmm* SFO model. In this way, we analyze a particular solution to the geometry optimization problem that can be considered a complementary approach to phonon frequency calculations. The advantage of the symmetry-mode analysis lies in the computation of relaxed supercell only using the DFT program without any imposed symmetry constraints. On the contrary, the phonon frequencies are calculated at each symmetry-allowed atomic displacement.

In the symmetry-mode analysis, static frozen distortions in a supercell are modes found by comparing the parent high-symmetry structure (SG *I*4/*mmm* model) and the distorted one (SG *P*1 model). Such modes are symmetry-adapted displacements fulfilling the symmetry properties of the supercell. The contribution of each symmetry allowed mode, $\tau$, to the distortion is given by the *global* amplitude $A_{\tau }$ which relates the atomic displacement $\textbf{u}(\mu ,i)$ for atom $\mu$ and its position splitting *i* in the distorted structure and normalized *polarization* vector $\mathbf {e(\tau )}$ by^39^

1 $$\begin{aligned} \textbf{u}(\mu ,i)=\sum _{\tau }A_{\tau }{} \textbf{e}(\tau |\mu ,i). \end{aligned}$$

To compare distorted structures of different sizes, we consider amplitudes, $A_{\tau }$, normalized within a primitive unit cell of the high-symmetry structure.

### Crystal symmetries

Following our approach described above, we consider SFO in two main different SG models taken as one basic *I*4/*mmm* (so far the only one suggested in the literature for SFO) and one new *Cmce* model (suggested in the presented study). The tetragonal *I*4/*mmm* (*tI*, $D_{4h}^{17}$, SG 139) model is considered to index experimentally obtained SFO structure from the ambient temperature down to 4 K in^25^ and 1.8 K in^41^. Here the primitive (*tP*) and body-centered (*tI*) tetragonal crystallographic bases

2 $$\begin{aligned}{}&\textbf{a}_{tI} = (a_0,0,0)^\text {T} \hspace{5.0pt}&\textbf{a}_{tP} =(-a_0/2,a_0/2,c_0/2)^\text {T} \nonumber \\&\textbf{b}_{tI} = (0,a_0,0)^\text {T} \hspace{5.0pt}&\textbf{b}_{tP} =(a_0/2,-a_0/2,c_0/2)^\text {T} \nonumber \\&\textbf{c}_{tI} = (0,0,c_0)^\text {T} \hspace{5.0pt}&\textbf{c}_{tP} =(a_0/2,a_0/2,-c_0/2)^\text {T} \end{aligned}$$

are connected with a transformation matrix $\varvec{Q}$

3 $$\begin{aligned} (\textbf{a}_{tI},\textbf{b}_{tI},\textbf{c}_{tI})=(\textbf{a}_{tP},\textbf{b}_{tP},\textbf{c}_{tP})\varvec{Q}, \hspace{5.0pt}\text {where} \hspace{5.0pt}\varvec{Q}= \begin{pmatrix} 0 &{} 1 &{} 1\\ 1 &{} 0 &{} 1\\ 1 &{} 1 &{} 0 \end{pmatrix}. \end{aligned}$$

**Figure 1:**
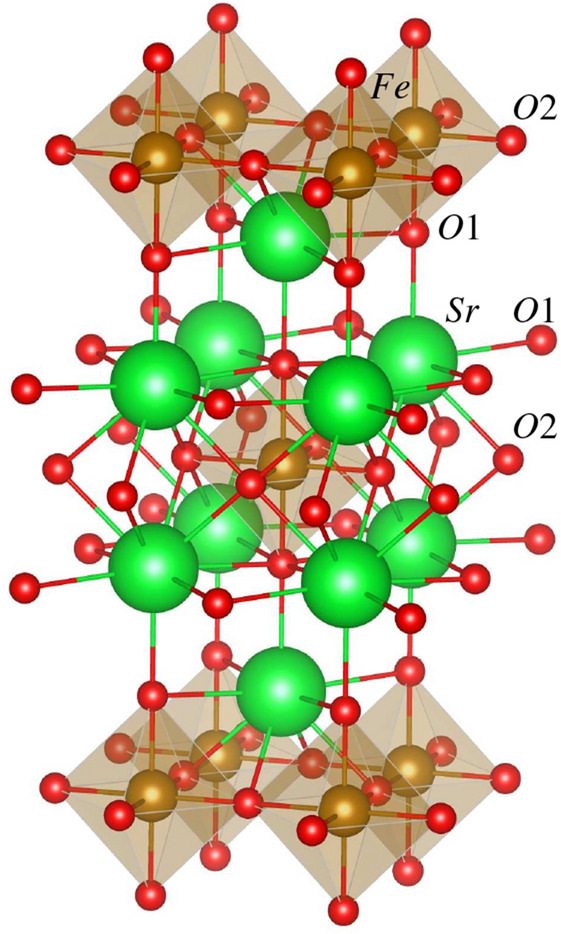
SFO in *I*4/*mmm* model. Sr atoms are green, Fe atoms are brown, and oxygen atoms are red. Oxygen octahedra around Fe atoms are shaded.

The ion Wyckoff positions (WP) and corresponding point symmetries in the crystallographic cell are given in Table 1. There are seven ions (one formula unit) in the primitive unit cell and 14 ions (two formula units) in the crystallographic unit cell. Therefore, the ion multiplicity is given for the crystallographic unit cell in Table 1. As expected, the two oxygens, O1 in the SrO-layer and O2 in the FeO$_{2}$-layer, occupy different WPs (Fig. 1). The oxygen in the SrO-layer (O1) is characterized by a higher point symmetry (point group C$_{4v}$) and free parameter $z_{O1}$ in comparison with the oxygen O2 (point group D$_{2h}$). The calculated lattice parameters $a_0$ and $c_0$, as well as free parameters $z_{Sr}$ and $z_{O1}$ for Sr and O1 WPs (4e), respectively, are given in Table 2 along with experimental lattice parameters taken from the literature.

**Table 1:** SFO in the *I*4/*mmm* and *Bbem* models.

Atoms	WP	Site symmetry	Representative position
*I*4/*mmm* (SG. 139)			
Sr	4e	4mm (C$_{4v}$)	(0,0,$z_{Sr}$)
Fe	2a	4/mmm (D$_{4h}$)	(0,0,0)
O1	4e	4mm (C$_{4v}$)	(0,0,$z_{O1}$)
O2	4c	mmm. (D$_{2h}$)	(0,1/2,0)
*Bbem* (SG. 64)			
Sr	8d	..2 (C$_{2}$)	(0,0,$z'_{Sr}$)
Fe	4a	..2/m (C$_{2h}$)	(0,0,0)
O1	8d	..2 (C$_{2}$)	(0,0,$z'_{O1}$)
O2	8f	..m (C$_{s}$)	($0.250{+}x'_{O2}$,$0.250{+}y'_{O2}$,0)

Wyckoff positions (multiplicity and Wyckoff letter), the corresponding oriented site symmetry (point group) and coordinates of the representative position in fractional units are given for each ion, respectively. The *Bbem* model allows a direct comparison of coordinates in the two models. We use all commonly accepted crystallographic notations for clarity and to avoid misinterpretations. Thus, the oriented site symmetry is given based on short point-group symbols of *International Tables*, while Schoenflies notation is given in parenthesis. Primed parameters $z'_{Sr}$ and $z'_{O1}$ in *Bbem* model are directly comparable with those of $z_{Sr}$ and $z_{O1}$ in *I*4/*mmm* ($x'_{O2}{=}y'_{O2}{=}0$ in *I*4/*mmm*) model.

**Table 2:** Comparison of calculated and experimental structural and electronic properties of SFO in the *I*4/*mmm* model.

Parameters	Calculated values			Experiments			
	WC1PW	WC3PW	PBE1PBE	1.8 K^41^	300 K^15^	4.2 K^25^	ambient^25^
$a_{0}$, Å	3.830	3.846	3.877	3.846	3.863	3.861	3.864
$c_{0}$, Å	12.282	12.324	12.393	12.374	12.391	12.399	12.397
$c_{0}/a_{0}$	3.207	3.204	3.197	3.217	3.208	3.211	3.208
$V_{0}$, Å$^3$	180.1	182.3	186.2	184.0	184.9	184.8	185.1
$z_{Sr}$	0.357	0.357	0.358	0.357	0.356	0.357	0.357
$z_{O1}$	0.156	0.156	0.156	0.159	0.158	0.157	0.157
$d_{Sr-O1}^{short}$, Å	2.468	2.477	2.496			2.481	2.475
$d_{Sr-O1}^{long}$, Å	2.713	2.725	2.747			2.736	2.738
$d_{Sr-O2}$, Å	2.595	2.605	2.621			2.620	2.623
$d_{Fe-O1(ap)}$, Å	1.921	1.927	1.936	1.967	1.963	1.948	1.950
$d_{Fe-O2(eq)}$, Å	1.915	1.923	1.938	1.928	1.932	1.931	1.932
$q_{Sr}$, $e^{-}$	+1.98	+1.98	+1.97				
$q_{Fe}$, $e^{-}$	+1.69	+1.70	+1.74				
$q_{O1}$, $e^{-}$	$-1.57$	$-1.56$	$-1.56$				
$q_{O2}$, $e^{-}$	$-1.26$	$-1.26$	$-1.29$				
$\mu _{Fe}$, $\mu _B$	3.62	3.64	3.68	$2.1{-}3.31/1.9{-}3.46$			
$E_g^{up}/E_g^{down}$, eV/eV	0/1.7	0/1.7	0/1.8				

Lattice parameters $a_0$, $c_0$ and their ratio, equilibrium volume $V_0$, effective atomic charges *q*, magnetic moments $\mu$, and band gaps in spin-up, $E_g^{up}$, and -down, $E_g^{down}$, channels, the distances *d* between cations and oxygens, as well as free parameters $z_{Sr}$, $z_{O1}$ for the Wyckoff position 4e (Table 1) are given. Notice that we distinguish two $d_{Sr-O1}$ distances (Fig. 1): ‘long’ due to O1 in the same SrO layer and ‘short’ due to O1 in the neighbouring SrO layer. Notice the experimental values for $\mu$ = 1.9-3.46 $\mu _B$ are taken from^15^.

**Figure 2:**
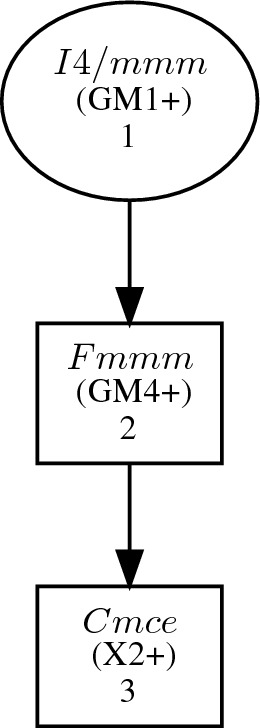
Isotropy subgroups between parent group *I*4/*mmm* and subgroup *Cmce* compatible with transformation matrix *P* (Eq. 4). The physically induced irreducible representations (irreps) in Cracknell–Davies–Miller–Love^42^ notations leading to the given subgroup are provided in brackets. Obtained using Get_irreps program of BCS^40^.

Our suggested SFO model represents a theoretically derived orthorhombic *Cmce* (*oS*, $D_{2h}^{18}$, SG 64) model (“Vibrational mode analysis”). SG *Cmce* is a subgroup of *I*4/*mmm* (Fig. 2), where new base-centred orthorhombic basis (*oS*) could be obtained by transforming the tetragonal crystallographic basis Eq. (2) using a transformation matrix $\varvec{P}$ without coordinate translation

4 $$\begin{aligned} (\textbf{a}_{oS},\textbf{b}_{oS},\textbf{c}_{oS})=(\textbf{a}_{tI},\textbf{b}_{tI},\textbf{c}_{tI})\varvec{P}, \hspace{5.0pt}\text {where} \hspace{5.0pt}\varvec{P}= \begin{pmatrix} 0 &{} 1 &{} \overline{1}\\ 0 &{} 1 &{} 1\\ 1 &{} 0 &{} 0 \end{pmatrix}. \end{aligned}$$

Crystallographic and primitive cells contain 28 and 14 atoms in the *Cmce* model, respectively, with the following WP splitting (Table 1)

5 $$\begin{aligned} {\begin{matrix} 4e &{}\rightarrow 8d \\ 4c &{}\rightarrow 8f \\ 2a &{}\rightarrow 4a. \end{matrix}} \end{aligned}$$

However, aligning the *z*-axis direction of the SG 64 standard *Cmce* model setting to the *I*4/*mmm* model is necessary to compare these models directly. This could be done by selecting non-standard setting *Bbem* of the SG 64 with the following basis vector exchange $(\textbf{b}_{oS},\textbf{c}_{oS},\textbf{a}_{oS})$. Below we refer to this setting only if not otherwise stated. We disregard the *Fmmm* group from further considerations since no displacive mode of this symmetry exists^43^.

Finally, we must select the supercell size for the triclinic *P*1 (*aP*, $C_1^1$, SG 1) model to apply the symmetry-mode analysis method. It should be at least as large as to accommodate a lower symmetry structure. As a reasonable starting guess, we construct the triclinic supercell model by doubling the size of *I*4/*mmm* crystallographic cell basis vectors $\textbf{a}_{tI}$ and $\textbf{b}_{tI}$ (Eq. 2)

6 $$\begin{aligned} (\textbf{a}_{aP},\textbf{b}_{aP},\textbf{c}_{aP})=(\textbf{a}_{tI},\textbf{b}_{tI},\textbf{c}_{tI})\varvec{N}, \hspace{5.0pt}\text {with} \hspace{5.0pt}\varvec{N}= \begin{pmatrix} 2 &{} 0 &{} 0\\ 0 &{} 2 &{} 0\\ 0 &{} 0 &{} 1 \end{pmatrix}. \end{aligned}$$

Hereafter, we call this supercell 2x2x1.

### Crystalline orbitals

To get a deeper insight into the mechanism of the Jahn-Teller effect from the chemical bond perspective, we perform a chemical bonding analysis based on the symmetry analysis of crystalline orbitals (COs). In the *I*4/*mmm* model of SFO, the Fe ion and its six nearest neighbouring (NN) oxygen ions form a metal-ligand FeO$_{6}$ complex with the D$_{4h}$ point group symmetry. Therefore, the dominating contributions to $\sigma$- and $\pi$-type COs come from the Fe 3*d* and O 2*p* electrons. In Mulliken notations, the Fe 3*d* atomic orbitals (AOs) transform according to the following irreducible representations (irreps): $\Gamma _{3d_{z^2}}=A_{1g}$ (where $z^2\equiv 2z^2-x^2-y^2$), $\Gamma _{3d_{x^2-y^2}}=B_{1g}$, $\Gamma _{3d_{xy}}=B_{2g}$, and $\Gamma _{3d_{xz,yz}}=E_g$.

On the other hand, the ligand orbitals formed by O1 and O2 2*p* AOs transform according to

7 $$\begin{aligned} \Gamma _{\sigma }= & {} 2A_{1g}+B_{1g}+A_{2u}+E_u, \end{aligned}$$

8 $$\begin{aligned} \Gamma _{\pi }= & {} A_{2g}+B_{2g}+2E_g+A_{2u}+B_{2u}+2E_u. \end{aligned}$$

For further analysis, it is convenient to link the particular oxygen AOs to their irreps in Eqs. (7, 8). Thus, the apex oxygen (O1) $2p_{\sigma }$ AO, that is directed towards Fe, transforms as $\Gamma _{2p_{\sigma }}=A_{1g}+A_{2u}$, while $2p_{\pi }$ AOs in the *xy* plane transform as $\Gamma _{2p_{\pi }}=E_g+E_u$ irreps. The equatorial oxygen (O2) 2$p_{\sigma }$ AO, that is directed towards Fe, transforms as $\Gamma _{2p_{\sigma }}=A_{1g}+B_{1g}+E_u$, the $2p_{\pi \Vert }$ AO in the *xy* plane transforms as $\Gamma _{2p_{\pi \Vert }}=A_{2g}+B_{2g}+E_u$, and the $2p_{\pi \perp }$ AO perpendicular to the *xy* plane transforms as $\Gamma _{2p_{\pi \perp }}=E_g+A_{2u}+B_{2u}$ irreps.

Finally, we construct the COs from Fe and oxygen ligand AOs of identical symmetry. In case of $\sigma$-based COs, the Fe:$3d_{z^2}$ bonds with both O1:$2p_{\sigma }$ and O2:$2p_{\sigma }$ (irrep $A_{1g}$) while Fe:$3d_{x^2-y^2}$ bonds only with O2:$2p_{\sigma }$ (irrep $B_{1g}$). Other Fe:$3d_{xy},3d_{xz},3d_{yz}$ AOs form no $\sigma$-based COs. In turn, $\pi$-based COs are formed by bonding Fe:$3d_{xy}$ and O2:$2p_{\pi \Vert }$ (irrep $B_{2g}$), while Fe:$3d_{xz,yz}$ bonds with both O1:$2p_{\pi }$ and O2:$2p_{\pi \perp }$ (irrep $E_{g}$). The Fe $3d_{z^2},3d_{x^2-y^2}$ AOs form no $\pi$-based COs.

## Simulation results

SFO does not differ from other similar transition metal oxides in the sense of careful choice and treatment of the DFT functional. It is widely accepted in the literature that conventional DFT functionals are unable to treat most of the properties of transition metal oxides (see, for example, excellent review paper of Cramer and Truhlar^44^ and Hasnip et al.^45^). For example, a famous ’bandgap problem’ is the most attractive motive for comparison studies between the density functionals. As conventional DFT functionals suffer from the self-interaction error, better methods to correct it need to be chosen. Among them, the hybrid DFT method based on a combination of Hartree-Fock exchange and conventional DFT functional lies at the heart of the present study. We compare three unique hybrid DFT functionals, namely WC1PW, WC3PW, and PBE1PBE, to elucidate what density functional is better to use in further analysis of symmetry reduction of SFO (“Density functionals”). The basic bulk properties are calculated for SFO in the *I*4/*mmm* model using all three hybrid DFT functionals. As a result, the hybrid WC3PW functional is the most consistent one by comparing the calculation results with the measured properties. Once the density functional is known, we can proceed to further calculations of SFO in different models and states.

### Ground state structure and meta-stable states

**Figure 3:**
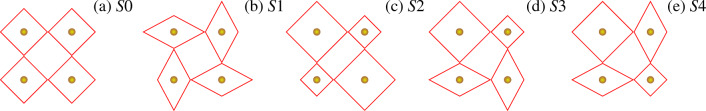
Schematic drawings of different FeO$_6$ octahedra relaxation patterns (top view of equatorial plane) in the FeO$_2$-layers in the original *I*4/*mmm* model (**a**) and in the 2 $\times$ 2 $\times$ 1 supercell in the *P*1 model: *S*1 (**b**), *S*2 (**c**), *S*3 (**d**) and *S*4 (**e**). Fe ions are yellow circles, while red lines represent connections between oxygens.

We used the 2 $\times$ 2 $\times$ 1 supercell obtained by applying a diagonal matrix *N* (Eq. 6) to the crystallographic unit cell for the evaluations of distortions in SFO. A complete symmetry switch-off (the *P*1 model) leads to 56 ions, from which 8 Fe ions are non-equivalent. An effective CRYSTAL geometry optimisation routine based on analytical gradients is well suited to relax such complex structures under conditions of complete symmetry switch-off. As a result, we obtain several metastable states with different relaxation patterns depending on the initial guesses. In other words, gradual symmetry reduction via subgroups of the *I*4/*mmm* SG is possible in CRYSTAL calculations, allowing us to obtain such relaxation patterns. We find four dominant structures denoted as *S*1, *S*2, *S*3 and *S*4 (Fig. 3) where the lowest total energy (ground state *S*1) structure appears to be energetically favourable by 121 meV/f.u. to the *I*4/*mmm* tetragonal one (*S*0) (see $\Delta E$ values in Table 4). The total energy difference between the other three structures (metastable states) and *S*0 is 44 (*S*2), 77 (*S*3), and 68 (*S*4) meV/f.u.

We find that the oxygen ions in the FeO$_{2}$-layer become unstable and move either closer or further away from the nearest Fe ion concertedly (Fig. 3). Interestingly, the relaxation pattern around Fe$^{4\texttt {+}}$ in the equatorial plane (FeO$_2$-layer) represents a square or rhombus, which is, consequently, reflected in the Fe magnetic moment ($\mu _{Fe}$), its effective atomic charge ($q_{Fe}$), and Fe-O-distances ($d_{Fe-O}$) (Table 3). There are three different sets of values for $d_{Fe-O}$, $q_{Fe}$ and $\mu _{Fe}$ in the *S*3 and *S*4 structures, as these structures contain three kinds of relaxation pattern: large square, small square and rhombus. The ground state structure (*S*1) contains only one kind of relaxation pattern, rhombus, and has all the Fe ions in the high spin state with no differences in their $\mu _{Fe}$-values. In the *S*1 structure, the FeO$_6$ octahedra are distorted to form a 90$^{\circ }$ rotated rhombuses (Fig. 3b). Interestingly, the orthorhombic structure (*S*1) has two different $d_{Fe-O}$ distances in the xy-plane (Table 3). One of the two distances in the xy-plane is largest among the three $d_{Fe-O}$ distances which is consistent with the calculations for K$_{2}$CuF$_{4}$ and Rb$_{2}$CuCl$_{4}$^34^. In a comparison with the tetragonal phase (*S*0), $\mu _{Fe}$-value of *S*1 is only slightly reduced, i.e. 3.64 vs 3.52 $\mu _{B}$. We suggest that ligand holes are distributed differently between the cations and oxygens due to different relaxation patterns in the negative charge transfer material like SFO leading to the $\mu _{Fe}$-values from 3.02 to 4.10 $\mu _{B}$. Also, the effective atomic charges ($q_{Fe}$) vary substantially depending on the relaxation pattern, i.e. from 1.56 to 1.79 $e^{-}$. Among these values, the small square pattern is characterised by the smallest values of $\mu _{B}$ and $q_{Fe}$, which also correlates with the smallest distances $d_{Fe-O}$. Due to its smallest charge, we attribute the small square pattern to an almost 3+ state. It is worth noting that the Fe-O distances of the 3+ state are smaller than those of the 4+ states in BaFeO$_{3}$^22^. Consequently, the large square pattern is attributed to the almost formal 4+ state. Thus, the square patterns represent extreme cases, while the rhombus pattern suggests an intermediate state. Finally, all the calculated structures are characterized by non-zero band gap values in the spin-up as well as spin-down channels (Table 4).

Notice that Supplementary Table S2 gives basic bulk properties for the ground state structure, whereas Fig. S1 demonstrates its band structure and partial density of states.

**Table 3:** Comparison of calculated structural and electronic properties of SFO in the *S*0, *S*1, *S*2, *S*3 and *S*4 structures (Fig. 3).

$d_{Fe -O1(ap)}$, Å	$d^{short}_{Fe -O2(eq)}$/$d^{long}_{Fe -O2(eq)}$, Å/Å	$q_{Fe}$, $e^{-}$	$\mu _{Fe}$, $\mu _B$	Octahedra shape
*S*0 structure (*I*4/*mmm* model)				
1.93	1.92/1.92	+1.70	3.64	Square
*S*1 structure (*P*1 model)				
1.92	1.82/2.04	+1.66	3.52	Rhombus
*S*2 structure (*P*1 model)				
1.97	1.98/1.98	+1.77	4.05	Large square
1.92	1.85/1.85	+1.58	3.08	Small square
*S*3 structure (*P*1 model)				
1.97	1.98/1.98	+1.78	4.06	Large square
1.92	1.83/2.03	+1.67	3.53	Rhombus
1.92	1.85/1.85	+1.58	3.06	Small square
*S*4 structure (*P*1 model)				
1.97	1.99/1.99	+1.79	4.10	Large square
1.93	1.85/2.00	+1.67	3.55	Rhombus
1.93	1.84/1.84	+1.56	3.02	Small square

Effective atomic charges $q_{Fe}$ and magnetic moments $\mu _{Fe}$ of Fe$^{4\texttt {+}}$ ions, the distances *d* between cations and oxygens are given.

### Jahn–Teller effect in Sr$_{2}$FeO$_{4}$

Let us consider the Jahn–Teller effect to explore the driving force for the observed symmetry lowering. In concentrated systems of Jahn-Teller ions, a cooperative distortion and orbital ordering, i.e., cooperative Jahn–Teller effect, may lead to the phase transition that lifts the orbital degeneracy^46,47^. Three types of interactions between Jahn–Teller ions could be distinguished: electronic-vibrational, quadrupole and exchange. The first two interactions are of Coulomb nature. Contrary to them, the third interaction depends on spin^46^.

In its classical formulation^48^, we consider only electronic and vibrational modes, where the degenerate ground, $\Gamma$, or close-in energy non-degenerate ground, $\Gamma$ and excited, $\Gamma '$, (pseudo-degenerate) electronic states could become unstable via suitable phonon mode, *Q*, (vibronic coupling) leading to the proper Jahn–Teller or pseudo-Jahn–Teller effects, respectively. The group-theoretical analysis of electronic and phonon modes predicts the nonzero energy terms

9 $$\begin{aligned}{} & {} \langle \Gamma | H'_Q| \Gamma \rangle \ne 0, \quad \text {if } \, \, \Gamma _Q \in \Gamma \times \Gamma , \end{aligned}$$

10 $$\begin{aligned}{} & {} \langle \Gamma | H'_Q| \Gamma '\rangle \ne 0,\quad \text {if }\,\, \Gamma _Q \in \Gamma \times \Gamma ', \end{aligned}$$

that could decrease high symmetry state energy only when the irreps fulfil the conditions (Eqs. 9 and 10) for the proper Jahn–Teller and pseudo-Jahn–Teller effects, respectively. In the following, we present a separate analysis of the vibrational and electronic modes and a discussion on their coupling.

#### Vibrational mode analysis

Group-theoretical methods are necessary for guiding us with a symmetry description of obtained distorted structures, particularly the one suggested as the ground state structure. In the group-theoretical analysis, the most relevant mode is identified and given by a specific irrep. Knowledge of this irrep and the movement of the corresponding ions specifies the Jahn–Teller effect in the DFT calculations. As a result, comparing the results of DFT calculations and experimental data is more effective and precise.

As is explained above, our calculations involve the 2 $\times$ 2 $\times$ 1 supercell calculated without symmetry constraints imposed. The obtained relaxed structure is analysed by the symmetry-mode method in AMPLIMODES program^38^. In the symmetry-mode analysis, the two structures are compared, i.e. the relaxed, low symmetry structure (obtained in the *P*1 model) and a reference high-symmetry structure (tetragonal SFO). The corresponding results from the collation of the two structures are collected in Table 4. In Table 4 A$_{Si}$ are said to be amplitudes in Eq. (1) for the ground state (*S*1) and mesta-stable states (*S*2, *S*3, *S*4), and, thus, represent the essential criterion for suggesting the irrep associated with the structure distortion. As is seen, the ground state structure *S*1 has only one value for the amplitude, namely 0.16 Å. The meta-stable states are characterised by contributions of one (*S*2), three (*S*3), and two (*S*4) amplitudes A$_{Si}$ varying from 0.03 to 0.11 Å, respectively. Each non-zero amplitude is associated with the specific irrep. Notice that the non-zero amplitudes of GM1+ are not associated with the symmetry change. The complete list of irreps and isotropy subgroups originating from the displacements of ions according to the symmetry-adapted displacive modes compatible with the symmetry break between the relaxed, low symmetry structure (SG *P*1 model) and high symmetry structure (SG *I*4/*mmm* model) are also added in Table 4.

Generally, relaxation according to a particular mode could lead to different space groups depending on the direction in the irrep space. In the *P*1 model, the X2+ ($b_{1g}$) is a secondary mode, resulting in the isotropic subgroup *Pbam* (SG 55) of *I*4/*mmm* (SG 139), since the symmetry-mode analysis provides the lowest symmetry estimate. However, after detailed inspection, one finds that the X2+ is a primary mode for transition to the *Cmce* (SG 64) subgroup (Table 4). We confirm that these two geometries are identical when comparing maximum atomic displacements, total distortion amplitude, energy gain, $\Delta E$, and geometry for the *Pbam* and *Cmce* models. A similar situation occurs with the X1+ ($a_g$) mode, which leads to the *Cmmm* model. Both models *Cmce* and *Cmmm* result in rhombus and large/small square structures (Fig. 3b, c) that can be described in a crystallographic cell with 28 atoms, respectively. Since our *P*1 model is larger (56 atoms in a crystallographic cell), we observe more metastable configurations as a result of the interplay among several modes X1+, X2+, and SM1 ($a_1$).

We also perform phonon calculations to analyse mode stability and provide only the smallest frequency for each irrep in Table 4. Phonon calculations confirm unstable modes obtained by the symmetry-mode analysis of different *P*1 model structures.

In supplementary, we provide a CIF file for SFO in the *Cmce* model optimised with the WC3PW functional. Moreover, differences in diffraction patterns between the *I*4/*mmm* and *Cmce* phases of SFO consist mainly of slight signal shifts at fixed intensity values (Fig. S3). In the region of $2\theta {\sim } 70^{\circ }$ for $\lambda {=}2.38$ Å and $2\theta {\sim } 6^{\circ }$ for $\lambda {=}0.2075$ Å, we observe the merging of two peaks in *I*4/*mmm* model into a one larger in *Cmce* model due to the shift of peaks in opposite directions.

**Table 4:** Amplimode analysis for four structures *Si* ($i{=}1$, 2, 3, 4) relative to the parent tetragonal structure (the *I*4/*mmm* model, $(\textbf{a}_{tI},\textbf{b}_{tI},\textbf{c}_{tI})$).

**K** vector	Irrep	$\omega _{min}$, cm$^{-1}$	Direction	IS	A$_{S1}$, Å	A$_{S2}$, Å	A$_{S3}$, Å	A$_{S4}$, Å
2x2x1 *P*1 (1) model $(2\textbf{a}_{tI},2\textbf{b}_{tI},\textbf{c}_{tI})$								
(0,0,0)	GM1+	209	(a)	*I*4/*mmm* (139)	0	0.02	0.01	0.01
(0,0,0)	GM5+	130	(a,b)	$P\overline{1}$ (2)	0	0	0	0
(0,0,0)	GM3-	225	(a)	*I*4*mm* (107)	0	0	0	0
(0,0,0)	GM4-	347	(a)	$I\overline{4}m2$ (119)	0	0	0	0
(0,0,0)	GM5-	175	(a,b)	*Cm* (8)	0	0	0	0
(1/2,0,0)	SM1	$-604$	(a,b,c,d)	*Pm* (6)	0	0	0.08	0.11
(1/2,0,0)	SM2	117	(a,b,c,d)	*Pc* (7)	0	0	0	0
(1/2,0,0)	SM3	117	(a,b,c,d)	*Pc* (7)	0	0	0	0
(1/2,0,0)	SM4	116	(a,b,c,d)	*Pm* (6)	0	0	0	0
(1/2,1/2,0)	X1+	$-441$	(a,b)	*Pmmm* (47)	0	0.09	0.05	0.03
(1/2,1/2,0)	X2+	$-1702$	(a,b)	*Pbam* (55)	0.16	0	0.09	0
(1/2,1/2,0)	X3+	137	(a,b)	*Pccn* (56)	0	0	0	0
(1/2,1/2,0)	X4+	127	(a,b)	*Pnnn* (48)	0	0	0	0
(1/2,1/2,0)	X2-	152	(a,b)	*Pmmn* (59)	0	0	0	0
(1/2,1/2,0)	X3-	154	(a,b)	*Pnnm* (58)	0	0	0	0
(1/2,1/2,0)	X4-	115	(a,b)	*Pccm* (49)	0	0	0	0
(1,1,1)	M1+	145	(a)	*P*4/*mmm* (123)	0	0	0	0
(1,1,1)	M5+	111	(a,b)	$P2_1/c$ (14)	0	0	0	0
(1,1,1)	M3-	150	(a)	*P*4/*nmm* (129)	0	0	0	0
(1,1,1)	M4-	346	(a)	$P4_2/nmc$ (137)	0	0	0	0
(1,1,1)	M5-	75	(a,b)	$P2_1/m (11)$	0	0	0	0
*Cmmm* (65) model $(\textbf{c}_{tI},\textbf{a}_{tI}{+}\textbf{b}_{tI},-\textbf{a}_{tI}{+}\textbf{b}_{tI})$								
(0,0,0)	GM1+		(a)	*I*4/*mmm* (139)		0.02		
(1/2,1/2,0)	X1+		(a,a)	*Cmmm* (65)		0.09		
*Cmce* (64) model $(\textbf{c}_{tI},\textbf{a}_{tI}{+}\textbf{b}_{tI},-\textbf{a}_{tI}{+}\textbf{b}_{tI})$								
(0,0,0)	GM1+		(a)	*I*4/*mmm* (139)	0			
(1/2,1/2,0)	X2+		(a,a)	*Cmce* (64)	0.16			
$E_g^{up}/E_g^{down}$, eV/eV					1.8/2.1	1.0/1.5	1.1/1.8	1.3/1.6
Maximum atomic displacement, Å					0.11	0.06	0.10	0.08
Total distortion amplitude, Å					0.16	0.10	0.13	0.11
$\Delta E$, meV					$-121$	$-44$	$-77$	$-68$

The mode amplitudes A$_{Si}$ and total distortion amplitude are normalized to the primitive unit cell of the *I*4/*mmm* model. The symmetry of each mode is characterized by the irreps of the *I*4/*mmm* group, the restricted direction within the irrep space and the resulting isotropic subgroup (IS). $\Delta E$ the energy difference for each obtained structure *Si* with respect to the *I*4/*mmm* model per formula unit and band gaps for spin up and down channels, $E_g^{up}$/$E_g^{down}$ obtained in the *P*1 model. The minimal frequency obtained from phonon calculations in the *P*1 model for each irrep is denoted by $\omega _{min}$, where a negative frequency means instability. Notice that AMPLIMODES programm gives the results in the standard settings, meaning the *Cmce* setting for the SG 64. However, we also use a non-standard *Bbem* setting for the SG 64 throughout the text (“Crystal symmetries”).

#### Electronic mode analysis

**Figure 4:**
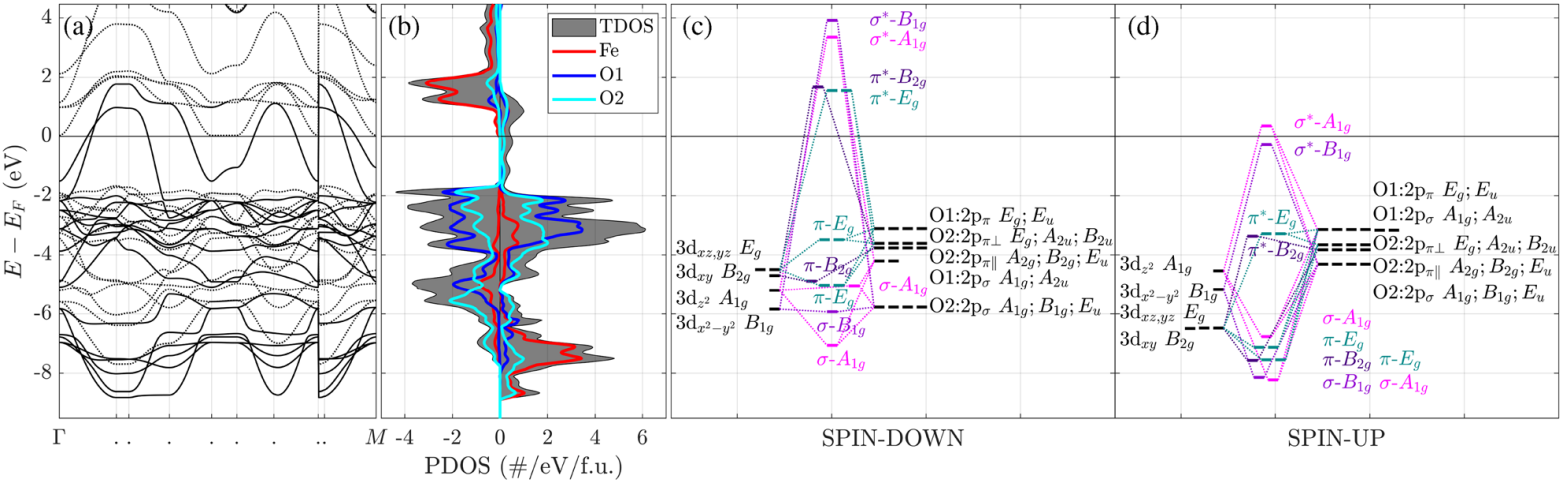
Band structure along high symmetry directions $\Gamma$-*X*-*P*-*N*-$\Gamma$-*M*-$S|S_0$-$\Gamma |X$-*R*|*G*-*M*^49^ in the Brillouin zone (**a**), the total (TDOS) and partial (atom projected) density of states (PDOS) (**b**) and CO diagrams (Fig. S3) for spin-down (**c**) and spin-up (**d**) electrons of SFO in the *I*4/*mmm* model. Spin-up and -down electrons correspond to solid and dotted lines in the band structure plot and positive and negative values of PDOS, respectively. Oxygen ligand orbitals $p_{\sigma }$ are directed towards Fe, the $p_{\pi }$ orbitals of O1 are two $\pi$ orbitals perpendicular to the Fe-O1 direction, while the $p_{\pi \Vert }$ orbitals of O2 are $\pi$ orbitals in the Fe-O2 layer perpendicular to the Fe-O2 direction, but the $p_{\pi \perp }$ orbitals of O2 are $\pi$ orbitals perpendicular to the Fe-O2 layer. All non-bonding E$_u$, A$_{2u}$, B$_{2u}$, and A$_{2g}$ orbitals are omitted for clarity. A thin solid line marks the Fermi level at 0 eV. For COs in colour, the corresponding irrep and type of bonding are given. The number of dashes is equivalent to the CO or AO degeneracy.

We calculated the band structure and partial density of states (PDOS) and extended our analysis to the properties and formation of COs. As is seen in the calculated band structure and DOS (Fig. 4), SFO is half-metallic in the *I*4/*mmm* model. It is reflected in (1) dispersed energy levels in the vicinity of the Fermi level at several high-symmetry k-points (Fig. 4a) and (2) the Fermi level crossing them in the spin-up channel. These energy levels are mainly given by the O 2*p* states hybridized with Fe 3*d* electrons (Fig. 4b). So, the same orbitals directly above the Fermi level represent expected hole states in the spin-up channel. The tail of dispersed O 2*p* states in the energy range between $-2$ and 0 eV is followed by the band of their even stronger interaction with the Fe 3*d* states at deeper energies. Accordingly, the Fe 3d states form a more localized band at approx. $-7$ eV from Fermi level beneath the O 2*p* band.

Moreover, the calculated Crystal Orbital Hamilton Population (COHP)^50,51^ allows us to analyse SFO electronic properties at the level of interacting AOs, thus giving deeper insight into the chemical bond formation. Namely, we refer to the CO diagrams for the FeO$_{6}$ octahedra and use corresponding nomenclature taking into account crystal symmetry properties (“Crystalline orbitals”). The local site symmetry of Fe$^{4\texttt {+}}$ ion in the tetragonal SFO (point group $D_{4h}$) ensures the contribution of 3*d* orbital projections to $B_{1g}$, $A_{1g}$, $E_{g}$, and $B_{2g}$ AOs (Fig. 4c, d). For example, the twofold degenerate $E_{g}$ AO of Fe$^{4\texttt {+}}$ is formed of $3d_{xz}$ and $3d_{yz}$ states, whereas $B_{2g}$ $(3d_{xy})$AO coincides with it in energy. Furthermore, one can analyse the O 2*p* (ligand) orbitals similarly.

As expected, the symmetry allowed Fe and ligand orbitals combinations led us to $\sigma$- and $\pi$-type bonding and anti-bonding COs. We, however, plot diagrams of COs without non-bonding states for the spin-up (Fig. 4d) and -down electrons (Fig. 4c), which is essential for the Jahn-Teller effect in SFO. We, first, emphasize the presence of the two non-degenerate anti-bonding COs in the vicinity of Fermi level in the spin-up channel, namely unoccupied $\sigma ^{*}{-}A_{1g}$ and occupied $\sigma ^{*}{-}B_{1g}$ COs. The splitting between the two is 0.63 eV, which is a bit smaller than in insulating K$_{2}$CuF$_{4}$^52^. We, second, emphasize a degenerate anti-bonding and occupied $\pi ^{*}{-}E_g$ orbital lying almost 3 eV lower and intermixed with a non-degenerate anti-bonding $\pi ^{*}{-}B_{2g}$ orbital. Essentially, removing its ($\pi ^{*}{-}E_g$) degeneracy provides grounds for the proper Jahn-Teller effect in SFO (see also discussion below). Both the apex O1 and equatorial O2 ions contribute to the formation of the anti-bonding $\pi ^{*}{-}E_{g}$ CO. Interestingly, there appear two bonding $\pi {-}E_{g}$ COs around ${-} 7$ eV given by the contributions from iron mainly. Similarly, $\sigma$-type orbitals analysis is done for the tetragonally distorted octahedral Mn$^{3\texttt {+}}$ in Ref.^53^.

Analysis of the COs for the spin-down electrons observes unoccupied anti-bonding $\pi ^{*}{-}E_{g}$ CO in contrast to the spin-up electrons. It correlates with the fact that the $E_{g}$ AO of Fe is higher in energy than the $B_{2g}$, $A_{1g}$ and $B_{1g}$ AOs in the spin-down channel. The analysis of COs with the inclusion of crystal symmetry is fundamental in explaining the FeO$_{6}$ octahedra distortions in SFO. These results give important hints to further similar studies on distortion mechanisms in perovskite oxides to discuss the Jahn-Teller effect therein properly.

**Figure 5:**
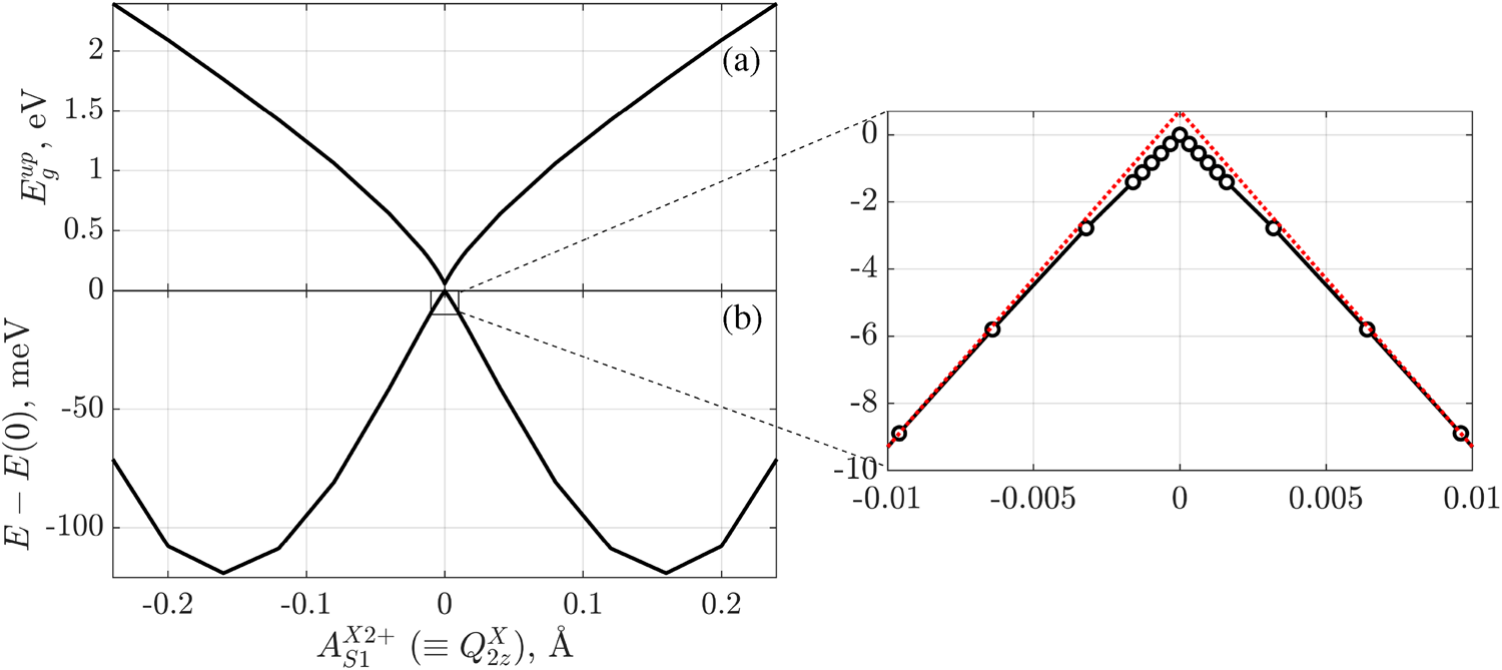
(**a**) Band gap in the spin-up channel and (**b**) adiabatic potential energy surface (APES) per SFO formula unit as a function of irrep X2+ displacement amplitude, $A^{X2\texttt {+}}_{S1}$ (equivalent to canonical label $Q^X_{2z}$^54^), in the *Cmce* model. The non-deformed, $A^{X2\texttt {+}}_{S1} {=} 0$, system’s energy, *E*(0), is selected as a reference state. Insert demonstrates the adiabatic potential energy in the $A^{X2\texttt {+}}_{S1}\rightarrow 0$ limit, where the tangents of the curve (red dotted lines) are added as a guide for the eye.

#### Discussion

Consider the condition Eq. (9) for the proper Jahn-Teller effect. The only degenerate electronic ground state in the $D_{4h}$ group is *E* leading to $[E \times E] = A_{1} + B_{1} + B_{2}$. Since the totally symmetric $A_1$ mode doesn’t lead to symmetry lowering, the proper Jahn-Teller effect is allowed via phonon mode $b_1$ or $b_2$ coupling, thus leading to the $E \otimes (b_1+b_2)$ problem^55^. A particular case, when the doublet weakly interacts with one of the vibrations, e.g., $b_2$, leads to the $E \otimes b_1$ problem^55^. Since our vibrational mode analysis demonstrates that the lowest energy structure *S*1 is obtained via X2+ (equivalent to $b_1$ in Mulliken notation), we conclude that the proper Jahn-Teller effect is allowed from a group-theory point of view.

To analyse conditions for the pseudo-Jahn–Teller effect (Eq. 10), we note that the highest occupied crystalline orbital (HOCO) is $B_{1g}$. Still, the lowest unoccupied crystalline orbital (LUCO) is $A_{1g}$, see Fig. 4(d). This leads us to $[A_1 \times B_1] = B_1$ estimate for electronic modes. Since X2+ mode ($b_1$) fulfils Eq. (10) condition, $b_1 \in B_1$, it follows that pseudo-Jahn-Teller effect is also allowed by group-theory. Vibrational modes X1+ and SM1 do not fulfil condition (Eqs. (9) and (10)) and thus lead to instabilities of different nature.

It is noted in Ref.^56^ that several types of Jahn-Teller effect can be present in the system simultaneously. They are, however, of different nature. Thus, in the proper Jahn-Teller case, there is a non-zero derivative of adiabatic potential energy surface (APES) at $Q{=}0$, leading to the nonzero driving force for high-symmetry structure distortion. In the pseudo-Jahn-Teller case, the APES derivative at $Q{=}0$ is zero, and the driving force of instability is added covalence^56^. Another important feature of the pseudo-Jahn-Teller effect is that only states with the same spin multiplicity could be coupled by vibronic modes^56^. In Fig. 5, we show how the APES and band gap in the spin-up channel depend on the Jahn–Teller instability mode $A^{X2\texttt {+}}_{S1}$: displacement of oxygens in the equatorial plane following the X2+ irrep. As expected, the APES has two equivalent minima for distortions in two directions with respect to the zero point. Based on this result, one can estimate the Jahn–Teller stabilization energy^57^, which is the same as the total energy difference $\Delta E$ in Table 4 and equals 121 meV/f.u. Besides, the present results are indicative of and consistent with the distortion of the $Q^{X}_{2z}$-mode type^54,58^. As is already discussed in Ref.^58^, the removal of an electronic $E_{g}$ orbital degeneracy is responsible for the existence of the Jahn–Teller effect and transformation to lower symmetries. Thus, SFO represents the case of such orthorhombic transformation following the X2+ irrep in the *I*4/*mmm* model or the $Q^{X}_{2z}$-mode in a more general language. Inspection of the APES dependence around $A^{X2\texttt {+}}_{S1} {=} 0$ confirmed the complexity of SFO and is essentially the reflection of the interplay between the proper and pseudo-Jahn-Teller effects. The band gap value decreases with the instability mode coordinate and is zero at $A^{X2\texttt {+}}_{S1} {=} 0$. We would like to emphasize that the X2+ irrep, which is the Jahn-Teller mode, should be accessible by Raman or similar experimental techniques.

SFO is complex because of its interacting magnetic, negative charge transfer, exchange and vibronic mode properties. The Jahn-Teller effect cannot be separated from and depends on all these properties. We hope that the resulting mechanisms described here give a sufficiently accurate picture to elucidate existing controversies and properly view this material and many other RP phases of the first order in the future.

## Conclusions

We suggest and explain conditions for a low symmetrical orthorhombic structure of SFO using group-theoretical analysis and hybrid DFT calculations. In particular, we use the symmetry mode and COHP analyses to find the space group of distorted structure and explain the underlying Jahn-Teller effect. Therefore, the vibrational and electronic modes are carefully analyzed. We define vibratrional instability modes as given by the X1+ ($a_g$), X2+ ($b_{1g}$) and SM1 ($a_1$) irreps. Moreover, the results of symmetry mode analysis coincide fully with those from the vibrational frequency calculations, as also done in the present study. The dominating X2+ mode is related to the bias of oxygen ions either closer or further away from the nearest Fe ion concertedly and crystal symmetry lowering down to orthorhombic SG *Cmce*. So, the FeO$_{6}$ octahedra are distorted in the equatorial plane with the formation of rhombuses, which is equivalent to the $Q^{X}_{2z}$-type^54^ Jahn–Teller mode, as discussed in the existing literature on perovskite systems.

Also, the same mode indicates the proper Jahn–Teller effect and $E \otimes b_1$ problem in a highly symmetrical SFO. However, the calculated APES indicates its complex nature. The presence of both proper Jahn–Teller and pseudo-Jahn–Teller effects is observed. The pseudo-Jahn–Teller effect is understood from the calculated crystalline orbitals $\pi ^{*}{-}A_{1g}$ and $\pi ^{*}{-}B_{1g}$ and their product $[A_1 \times B_1] = B_1$. Furthermore, it is established that (1) the three-parameter hybrid WC3PW functional, which combines the semi-local WC exchange and PW correlation with the parameter of 0.16 for the exact exchange part, gives the most accurate description of tetragonal SFO (SG *I*4/*mmm* model); (2) symmetry-mode analysis is sufficiently effective as it only requires accurate lattice parameters and optimised coordinates of atoms, which is advantageous compared to the phonons calculations; (3) CRYSTAL calculations using the analytic gradients are sufficiently accurate to perform the lattice parameters and atoms coordinates optimisation in a structure without imposing symmetry constraints; it is essential for the symmetry-mode analysis and distorted structure space group identification; (4) the COs need to be considered to properly discuss the mechanism of Jahn–Teller distortion in systems with holes and strong hybridisations between the transition metal and ligand states.

## Methods

### Calculation parameters

We perform the first-principles calculations within the DFT formalism using CRYSTAL23^37,59^ computer code. In CRYSTAL, the single-particle wave functions are expanded as a linear combination of Bloch functions defined in terms of atomic orbitals (LCAO), which, in turn, are a linear combination of Gaussian-type functions.

We use $20{\times }20{\times }20$, $5{\times }5{\times }7$, and $5{\times }5{\times }3$ Monkhorst-Pack *k*-point meshes^60^ for the *I*4/*mmm*, *Cmce*, and *P*1 models, respectively. An increased mesh density for the *I*4/*mmm* model is used since SFO demonstrates a half-metallic behaviour. The SCF convergence threshold for the total energy is set to $10^{-10}$ Hartree^59^. Although the system’s geometry is converged and could be qualitatively analysed at lower calculation accuracy, the higher calculation accuracy is chosen in this paper due to vibrational calculations. In particular, integration is performed on a predefined pruned grid consisting of 99 radial and a maximum of 1454 angular points (XXLGRID); DFT density and grid weight tolerances are 10 and 20, respectively.

Complete geometry optimisation is performed until the energy difference between two steps is less than the threshold (TOLDEE) $10^{-10}$ Hartree, the root-mean-square of the gradients (TOLDEG), and the estimated displacements (TOLDEX) are 0.00003 Hartree/Bohr and 0.00012 Bohr, respectively, using no trust radius to limit displacement (NOTRUSTR). For an accurate comparison of system energies of two different geometries, we use the FIXINDEX option^59^. The tolerances for Coulomb and exchange sums (five TOLINTEG parameters) are set to 10 10 10 10 20, respectively.

In simulations, we use optimised all-electron custom-made basis sets for Sr:24*s*17*p*8*d*1*f*/5*s*5*p*3*d*1*f*, Fe:20*s*14*p*5*d*1*f*/5*s*4*p*3*d*1*f*, O1 and O2:15*s*6*p*1*d*/4*s*3*p*1*d*. Here we provide the total number of Gaussian primitives/the number of contracted and uncontracted orbitals for each shell type. During the optimisation of O1 and O2 basis sets, we find that the O2 basis set is more localised than the O1 basis set (Table S1).

### Density functionals

In our calculations (“Calculation parameters”), we use hybrid functionals that, in the general form suggested by Becke in^61,62^, can be re-written as^59^

11 $$\begin{aligned} \begin{aligned} E_{xc} = E_x^{L(S)DA} + A \, (E_x^{HF}-E_x^{L(S)DA}) + (1-A)B \, (E_x^{DFA}-E_x^{L(S)DA}) + E_c^{L(S)DA}+C \, (E_c^{DFA}-E_c^{L(S)DA}) , \end{aligned} \end{aligned}$$

where $E_{x,c}^{L(S)DA}$ the local density functional exchange and correlation contributions and $E_{x,c}^{DFA}$ the semi-local density functional exchange and correlation contributions (such as GGA). The non-local density functional exchange and correlation contributions are given by *B* and *C* parameters, respectively, whereas *A* finds the amount of exact Hartree-Fock exchange $E_{x}^{HF}$.

Three functionals are defined in our calculations for different combinations of exchange and correlation parts. So, two functionals without the L(S)DA non-local part are obtained from Eq. (11) using $B{=}1$ and $C{=}1$

12 $$\begin{aligned} E_{xc} = A \, E_x^{HF} + (1-A) \, E_x^{DFA} + E_c^{DFA}, \end{aligned}$$

where for the exchange, $E_x^{DFA}$, and correlation, $E_c^{DFA}$ parts, we apply the following two combinations: $E_x^{DFA}$=$E_x^{WC}$ and $E_c^{DFA}$=$E_c^{PW}$ (combination1) and $E_x^{DFA}$=$E_x^{PBE}$ and $E_c^{DFA}$=$E_c^{PBE}$ (combination2) by analogy with the B1WC^63^ and PBE0^64^ hybrid exchange-correlation functionals, respectively. For the third functional based on Eq. (11), we considered the exchange and correlation contributions as $E_x^{DFA}$=$E_x^{WC}$, $E_c^{DFA}$=$E_c^{PW}$ and parameters $B{=}0.90$, $C{=}0.81$ (combination3) by analogy with Becke’s three-parameter functional B3PW^61^. Thus, the three functionals are denoted as WC1PW (combination1), PBE1PBE (combination2) and WC3PW (combination3) to reflect the used combinations and for consistency with accepted nomenclature in the literature.

The amount of exact HF exchange, *A*, for all the three functionals is determined self-consistently^59,65^ in the *Cmce* model of SFO. Calculations yield similar estimates of $A{=}16$% in all cases. Therefore, only this amount of exact HF exchange is used throughout the paper.

### Comparison of hybrid DFT functionals

One must obtain sufficiently accurate relaxed crystal structures to use the symmetry-mode analysis. Therefore, we compare three hybrid density functional (WC1PW, WC3PW and PBE1PBE) predictions with the experimental values from the literature (Table 2). It is essential that both these experiments (Refs.^25,41^), also containing low-temperature data, show weak SFO structure parameter dependence on temperature over a wide interval. Thus, the low-temperature experimental structure parameters may be directly compared with the first-principles calculations results at 0 K.

The WC^66^ exact exchange in either combination provides values smaller than those due to the PBE1PBE functional. The calculation results based on the WC1PW functional underestimate the SFO lattice parameters, including the equilibrium volume and the cation-oxygen distances compared to the experimental values (Table 2). However, the other two density functionals compete more. Lattice parameters $a_{0}$ and $c_{0}$ are better reproduced by WC3PW and PBE1PBE, respectively. In addition, the WC3PW functional gives a very good result for the ratio $c_{0}$/$a_{0}$=3.20 vs 3.22^41^, 3.21^25^. Both functionals, WC3PW and PBE1PBE, suggest comparable results for the equilibrium volume $V_{0}$ as it is by  2 Å$^3$ smaller for WC3PW but larger for PBE1PBE compared to the experimental value. All three functionals agree pretty well with each other and with the experiments on the comparison of free parameters of 4e WP: $z_{Sr}$=0.36 Å, $z_{O1}$=0.16 Å (Table 2). However, the inter-ionic distances are again sensitive to the functional choice. It is worth noting that we distinguish two Sr-O distances in the *I*4/*mmm* model. The short Sr-O distance, i.e. $d_{Sr-O1}^{short}$, is between Sr and oxygen O1 in the neighbouring SrO layer (Fig. 1) and is better reproduced by the WC3PW functional compared to the experiments. The long Sr–O distance, i.e. $d_{Sr-O1}^{long}$, is between Sr and O1 in the same SrO layer and is also well comparable with the experiments, if calculated by either WC3PW or PBE1PBE. However, the PBE1PBE functional gives the distance between Sr and O2 (Fig. 1, Table 2), i.e. 2.62 Å coinciding with the experimental value. Finally, the inter-ionic distances in the FeO$_6$ octahedra have also been analysed. In contrast to the PBE1PBE, the WC3PW functional predicts a slightly stretched FeO$_{6}$ octahedra along the *z*-axis, i.e. the relation between the corresponding inter-ionic distances $d_{Fe{-}O1(ap)}{>}d_{Fe{-}O2(eq)}$ is also in agreement with the experiments.

The three functionals do not disagree significantly with each other on the electronic properties. So, the effective atomic charges *q* are very close for Sr and O1. Nevertheless, these are slightly larger and more negative if calculated for Fe and O1 using the PBE1PBE functional compared to the WC functionals. In their interactions with oxygens, all three functionals agree on a covalent and ionic bonding for Fe and Sr. It is also reflected in more negative effective charges of O1 than O2. However, one needs to look more carefully at the magnetic moments $\mu _{Fe}$. The calculated values of $\mu _{Fe}$ (Table 2) such as 3.64 $\mu _{B}$, indicate Fe in the 4+ oxidation and high spin states. Notice that the formal value for $\mu _{Fe}$ in the high spin state ($t_{2g}^3e_g^1$) is 4. In recent magnetic experiments^15,41^, it is established that SFO adopts a cycloidal elliptical spin spiral magnetic structure below $T_{N}$ with magnetic moments modulation from 1.9 to 3.5 $\mu _B$. We consider the present hybrid DFT calculated values of $\mu _{Fe}$ to be quite consistent with the experimental values, taking into account our simplified model for magnetism.

Overall, we conclude that WC3PW is the most suitable functional for further calculations and analysis of distortion patterns. In supplementary, we provide a CIF file for SFO in the *I*4/*mmm* model optimised with WC3PW. In the following, all the results belong to this functional only.

### Supplementary Information

Supplementary Information.

## Data Availability

We provide CIF and CRYSTAL output files for SFO geometries in *I*4/*mmm* and *Cmce* models optimised with WC3PW functional in Novel Materials Discovery (NOMAD) Lab repository. Additional information is given in the Supplementary Information.
