# Supplementary material for: Jahn–Teller distortion in Sr2FeO4: group-theoretical analysis and hybrid DFT calculations
Source: Sci Rep. 2023 Sep 30;13:16446. doi: 10.1038/s41598-023-43381-7 (PMC10542785; doi:10.1038/s41598-023-43381-7)
Supplement: Supplementary file 1 — Supplementary Information. [file 41598_2023_43381_MOESM1_ESM.pdf]

Jahn-Teller distortion in Sr_2FeO_4: group-theoretical analysis and hybrid DFT calculations

Guntars Zvejnieks [1*], Yuri Mastrikov [1], and Denis Gryaznov [1*]

[1]Institute of Solid State Physics, University of Latvia, Kengaraga Str. 8, Riga, LV-1063, Latvia
[*]Authors to whom correspondence should be addressed: guntars.zvejnieks@cfi.lu.lv or denis.gryaznov@cfi.lu.lv

Table S1. Optimised all-electron basis sets for Sr_2FeO_4, where oxygen basis sets 8 and 108 correspond to O1 and O2 oxygens in Table I of the main text, respectively

Sr:24*s*17*p*8*d*1*f*/5*s*5*p*3*d*1*f*		Fe:20*s*14*p*5*d*1*f*/5*s*4*p*3*d*1*f*	
38 14		26 13	
0 0 9 2 1		0 0 9 2 1	
1437272.9559718600	0.0000334929	605314.2371399590	0.0000604070
214923.5486989340	0.0002612690	90614.4261434691	0.0004697121
48819.7447675914	0.0013697175	20618.4734371643	0.0024585116
13810.6913375600	0.0057101826	5826.8746437636	0.0102487810
4475.3438465298	0.0200300363	1886.2665682828	0.0358137796
1603.9945340342	0.0568126263	670.9818077505	0.1013326129
621.0228241221	0.1217684281	257.2207791189	0.2081185473
251.9975687297	0.1558826935	104.4105203171	0.2381288311
104.1244344459	0.0701776403	43.6246669728	0.0907224732
0 0 5 2 1		0 0 5 2 1	
1636.9789715162	-0.0062290974	738.3075208721	-0.0030417914
521.0529194409	-0.0467685960	238.1821619191	-0.0216917797
205.2928179810	-0.1366139908	93.5033436548	-0.0644954205
36.7659903038	0.7013131610	16.0583041623	0.3405540023
15.3186107714	0.6169458927	6.5030511322	0.3101608931
0 0 5 2 1		0 0 4 2 1	
174.7154873015	0.0019912308	107.6584868784	0.0024410802
51.2646156893	-0.0333816272	22.8254313403	-0.0221794412
23.3604846899	-0.3250890840	9.7459694074	-0.2514698836
4.6960331577	0.9747325618	1.8001837526	0.7540843263
2.0945392208	0.6062180404	0 0 1 0 1	
0 0 4 2 1		0.7489557333	1
25.0063148583	-0.0558022743	0 0 1 0 1	
6.9233880394	-0.0185402847	0.1982108765	1
2.5384236293	-0.4112041615	0 2 7 6 1	
0.6684529296	1.6617104988	3430.3198713250	0.0015999639
0 0 1 0 1		822.0450592566	0.0135324718
0.2863885108	1	263.6241802534	0.0736645839
0 2 7 6 1		98.7030779674	0.2648022213
7440.5258355798	0.0011317552	40.5047631360	0.6597302512
1773.1616684861	0.0096988572	17.4032147130	0.9782486916

568.8141240344	0.0518667592
214.3802720450	0.1887424489
88.9618427348	0.4607470538
38.6459718251	0.6510343394
16.7583876079	0.3504969357
0 2 5 6 1	
661.6276122151	-0.0025117708
172.7803792645	-0.0242354654
60.8579648099	-0.1018783628
10.1541071859	0.9921751679
4.3740452831	1.8312735338
0 2 3 6 1	
11.3662198041	-0.0121764268
4.6969921074	-0.0806827150
1.8000752931	0.1683042229
0 2 1 0 1	
0.7571926018	1
0 2 1 0 1	
0.2946114181	1
0 3 6 10 1	
405.6603368913	0.0045946357
121.0223572366	0.0382814494
46.1117192925	0.1699440444
19.3650216461	0.4580911071
8.5455016025	0.7431666641
3.7737725750	0.7087341867
0 3 1 0 1	
1.5832178133	1
0 3 1 2 1	
0.4425624950	1
0 4 1 0 1	
0.5170039898	1

7.5173256814	0.6067552158
0 2 5 6 1	
660.2430296325	0.0009173937
145.5439831265	0.0059287065
45.7365180915	0.0088035408
6.5740129476	0.1266816256
2.8232242213	0.6174369047
0 2 1 0 1	
1.1135089772	1
0 2 1 0 1	
0.3756206013	1
0 3 4 8 1	
94.3159654865	0.0150719242
26.5443553324	0.1179204903
9.0938093780	0.4488680766
3.3968689572	0.9579840000
0 3 1 0 1	
1.2556330043	1
0 3 1 0 1	
0.4167001504	1
0 4 1 0 1	
0.5025390600	1

O1:15s6p1d/4s3p1d

8 8	
0 0 9 2 1	
65995.8959523781	0.0001223379
9867.5448499900	0.0009407848
2237.3138855365	0.0050078502
630.6835837708	0.0209500039
204.1604514141	0.0727465362
73.0245876843	0.2075654601
28.0385610564	0.4448471530
11.3055599730	0.6023817452
4.5718014576	0.3417364130
0 0 4 2 1	
69.6736997532	-0.0088492753
19.7148843107	-0.0748886676
6.9547733451	-0.2063103072
1.2059865567	0.9167838489
0 0 1 0 1	
0.4268155172	1
0 0 1 0 1	
0.1499404420	1
0 2 4 4 1	

O2:15s6p1d/4s3p1d

108 8	
0 0 9 2 1	
67628.9919520143	0.0001190572
10111.7213671537	0.0009214347
2292.6771517469	0.0048865159
646.2901123713	0.0205040668
209.2124870245	0.0712259683
74.8316115955	0.2045082076
28.7323869563	0.4395436129
11.5853207747	0.6111442149
4.6849325935	0.3550680374
0 0 4 2 1	
73.7919987101	-0.0074340916
21.0321987423	-0.0710956392
7.2292304497	-0.2182350259
1.2320875359	0.8734442109
0 0 1 0 1	
0.4666263766	1
0 0 1 0 1	
0.1684758235	1
0 2 4 4 1	

	80.9326864908	0.0033044491		91.0092665016	0.0030938065
	18.4396020435	0.0256110083		20.7256283322	0.0242203033
	5.6012012154	0.1000338039		6.3740407624	0.0964645877
	1.9204759001	0.2483683241		2.1864314779	0.2574298403
0 2 1 0 1			0 2 1 0 1		
	0.6494722737	1		0.7464975296	1
0 2 1 0 1 0			2 1 0 1		
	0.1817231583	1		0.2184300050	1
0 3 1 0 1			0 3 1 0 1		
	0.1016862127	1		0.1432941233	1

References

1. Hinuma, Y., Pizzi, G., Kumagai, Y., Oba, F. & Tanaka, I. Band structure diagram paths based on crystallography. *Comput. Mater. Sci.* **128**, 140–184, DOI: https://doi.org/10.1016/j.commatsci.2016.10.015 (2017).

2. Hoffmann, R. How chemistry and physics meet in the solid state. *Angew. Chem. Int. Ed.* **26**, 846–878, DOI: https://doi.org/10.1002/anie.198708461 (1987).

3. Dronskowski, R. *Computational Chemistry of Solid State Materials* (John Wiley & Sons, Ltd, 2005).

4. Momma, K. & Izumi, F. *VESTA3* for three-dimensional visualization of crystal, volumetric and morphology data. *J. Appl. Cryst.* **44**, 1272–1276, DOI: https://doi.org/10.1107/S0021889811038970 (2011).

Table S2. Theoretically calculated parameters of SFO in the non-standard *Bbem* model (SG 64) setting using the WC3PW functional. For easier comparison, we align the z-axis direction of the standard *Cmce* model (SG 64) setting to the $I4/mmm$ model. Primed parameters are given for pseudo-tetragonal cell, and thus they are directly comparable with $I4/mmm$ model of SFO, where x'_{O2}=y'_{O2}=0

Parameters	Calculated values
a'_0=$b_{oS}/\sqrt{2}$, Å	3.863
b'_0=$c_{oS}/\sqrt{2}$, Å	3.864
c'_0=a_{oS}, Å	12.282
c'_0/a'_0	3.179
V'_0, Å^3	183.3
z'_{Sr}	0.357
z'_{O1}	0.156
(x'_{O2},y'_{O2})	(0.015,0.015)
q_{Sr}, e^-	1.98
q_{Fe}, e^-	1.66
q_{O1}, e^-	-1.56
q_{O2}, e^-	-1.25
μ_{Fe}, μ_B	3.52

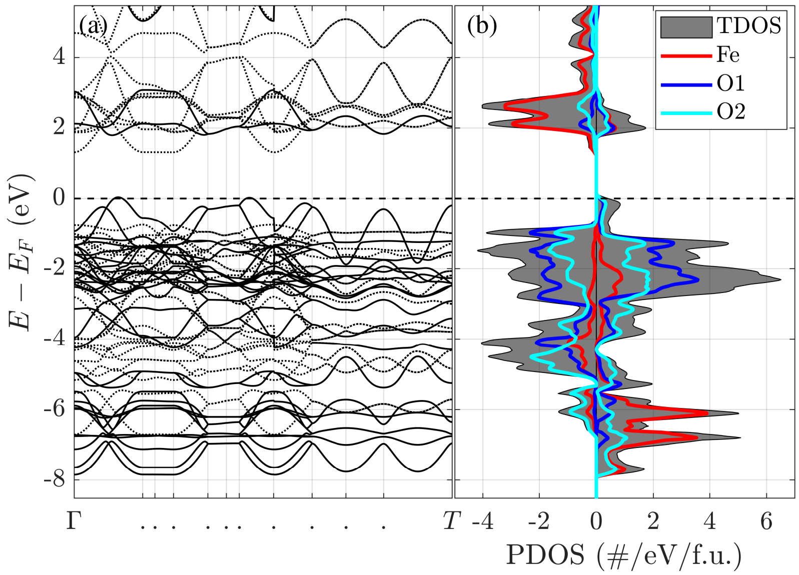

Figure S1. Band structure along high symmetry directions Γ-Y-$F_0|\Delta_0$-Γ-Z-$B_0|G_0$-T-$Y|\Gamma$-S-R-Z-T[1] in the Brillouin zone (a), the total (TDOS) and partial (atom projected) density of states (PDOS) (b) for SFO in *Cmce* model.

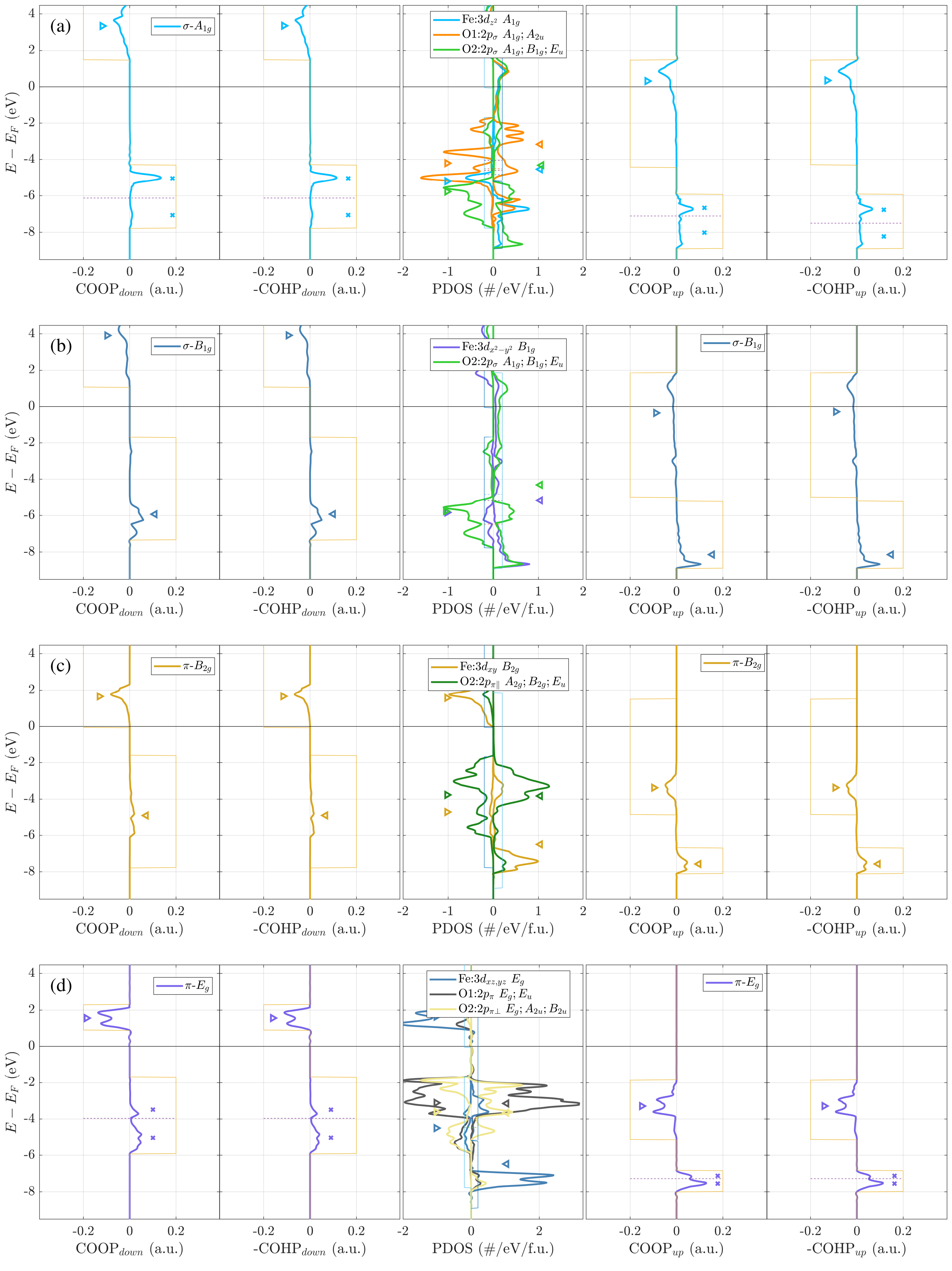

Figure S2. PDOS analysis of AOs, where spin-up and -down electrons correspond to positive and negative values, respectively. COOP and -COHP[2,3] analysis of COs, where bonding and anti-bonding intervals correspond to positive and negative values, respectively. Orbitals are distingueshed according to (a) A_{1g}, (b) B_{1g}, (c) B_{2g}, and (d) E_g irreps of $I4/mmm$ model. A triangle gives the centre of mass if a single maximum is expected in the integration interval (marked by a box) and crosses if two maxima are expected according to the group theory. Only the centres of mass from -COHP analysis are used for the COs diagram [Fig. (5) in the main text].

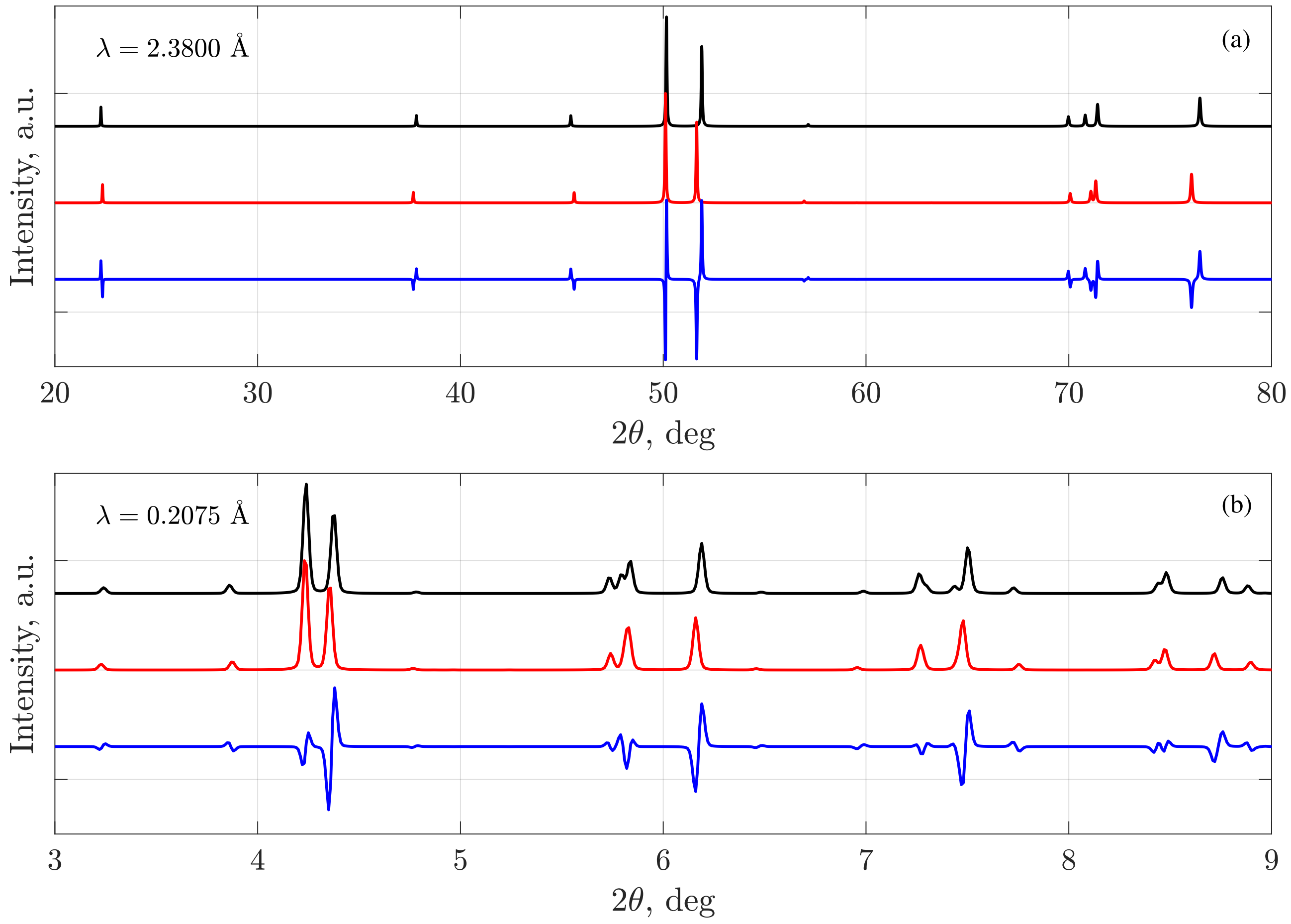

Figure S3. Diffraction pattern comparison for *I*4/*mmm* (black), *Cmce* (red) models and their difference (blue) for two characteristic wavelengths (a) λ=2.3800 and (b) λ=0.2075 Å. Generated with powder diffraction pattern tool of VESTA[4].
